# Weighted Interaction SNP Hub (WISH) network method for building genetic networks for complex diseases and traits using whole genome genotype data

**DOI:** 10.1186/1752-0509-8-S2-S5

**Published:** 2014-03-13

**Authors:** Lisette JA Kogelman, Haja N Kadarmideen

**Affiliations:** 1 Department of Veterinary Clinical and Animal Sciences, Faculty of Health and Medical Sciences, University of Copenhagen, Grønnegårdsvej 7, 1870 Frederiksberg C, Denmark

## Abstract

**Background:**

High-throughput genotype (HTG) data has been used primarily in genome-wide association (GWA) studies; however, GWA results explain only a limited part of the complete genetic variation of traits. In systems genetics, network approaches have been shown to be able to identify pathways and their underlying causal genes to unravel the biological and genetic background of complex diseases and traits, e.g., the Weighted Gene Co-expression Network Analysis (WGCNA) method based on microarray gene expression data. The main objective of this study was to develop a scale-free weighted genetic interaction network method using whole genome HTG data in order to detect biologically relevant pathways and potential genetic biomarkers for complex diseases and traits.

**Results:**

We developed the Weighted Interaction SNP Hub (WISH) network method that uses HTG data to detect genome-wide interactions between single nucleotide polymorphism (SNPs) and its relationship with complex traits. Data dimensionality reduction was achieved by selecting SNPs based on its: 1) degree of genome-wide significance and 2) degree of genetic variation in a population. Network construction was based on pairwise Pearson's correlation between SNP genotypes or the epistatic interaction effect between SNP pairs. To identify modules the Topological Overlap Measure (TOM) was calculated, reflecting the degree of overlap in shared neighbours between SNP pairs. Modules, clusters of highly interconnected SNPs, were defined using a tree-cutting algorithm on the SNP dendrogram created from the dissimilarity TOM (1-TOM). Modules were selected for functional annotation based on their association with the trait of interest, defined by the Genome-wide Module Association Test (GMAT). We successfully tested the established WISH network method using simulated and real SNP interaction data and GWA study results for carcass weight in a pig resource population; this resulted in detecting modules and key functional and biological pathways related to carcass weight.

**Conclusions:**

We developed the WISH network method which is a novel 'systems genetics' approach to study genetic networks underlying complex trait variation. The WISH network method reduces data dimensionality and statistical complexity in associating genotypes with phenotypes in GWA studies and enables researchers to identify biologically relevant pathways and potential genetic biomarkers for any complex trait of interest.

## Background

High-throughput genotype (HTG) data has been intensively used in genetic and genomic studies to unravel the genetic background and control mechanisms of complex diseases and traits in humans, plants, animals and many other organisms. As the genotyping costs decrease rapidly, HTG data is a favourable source of data to collect on a routine basis in many research, development and innovation industries. The main method used to identify genes associated with the disease or trait of interest has been genome-wide association (GWA) studies. However, the published GWA studies have, so far, typically identified genes with a low relative genotype risk and overall results only explain a small part of the predicted heritability [1]. The GWA study is a single-step approach, which means that each single nucleotide polymorphism (SNP) is tested with the trait of interest. This test is repeated for all SNPs on the SNP Chip, which causes the multiple-testing problem and subsequently, very stringent cut-off values result in many biologically relevant SNPs being missed [2]. Another, potentially larger, problem is the failure to explain entire underlying genetic variation in complex diseases and traits due to its inability to fit genome-wide genetic interactions [3]. Several studies have stated the importance of the interaction effects between genes, especially in complex diseases and traits [4]. By including the detection of gene *x *gene interactions in GWA studies, the power to detect biologically relevant SNPs is increased [5]. Several studies have detected the gene *x *gene interactions in two-stage models [6-8]; however, only two SNPs are taken into account at a time. Analysing many more than two SNP combinations in one model results in an exponential increase of the sample size needed, referred to as the curse of dimensionality [9]. In conclusion, sample size requirements and statistical-computational limitations increase the need for a method (such as WISH) which reduces the multiple-testing problem and takes interaction effects into account when analysing HTG data.

Systems genetics approaches focus on the network of interactions between genes and phenotypes in order to understand the complexity of diseases and traits [10,11]. Several approaches have been used to distinguish networks, functional pathways, and underlying causal genes to unravel the biological and genetic background of complex diseases [12-16]. Often, gene interaction occurs by the influence of several genes on a specific protein, resulting in (de-)activation of the protein. As we know that a particular phenotype is the result of the presence or level of expression of several genes, it is beneficial to understand how gene networks exist and behave under specific circumstances. The knowledge about pathways in the gene networks could help us to improve our understanding of the complexity of diseases and complex traits [17]. However, the relationship between genes and the disease or trait is often not straightforward. As several genes and their interactions result in a disease in one individual, a (partial) different set of genes may lead to the same disease in another individual. By defining modules and pathways and relating them to various diseases or trait of interest (endophenotypes [18,19]), a better biological understanding of complex diseases and traits would be possible. In addition, focusing on SNP modules and their pathways instead of single SNP effects is a network-based systems genetics technique which will alleviate the multiple-testing problem of GWA studies and consequently increase the power to detect biologically relevant genes.

Using microarray gene expression data, Langfelder and Horvath [20] proposed the Weighted Gene Co-expression Network Analysis (WGCNA) method, whereby the pairwise Pearson's correlation between gene expression values reveals the interaction between genes. The main assumption of WGCNA is that genes with similar expression patterns in a number of individuals are interconnected. Detection of genes that are highly interconnected will result in detection of gene modules which potentially represent biological pathways. WGCNA has been extensively used and has proven ability to identify pathways and potential genetic biomarkers in several traits and complex diseases [21-25]. WGCNA has also been compared to other category of scale-free network approaches in the context of its ability to retain important gene modules and detect biologically relevant hub genes and biomarkers. Results show that WGCNA is superior to other scale-free approaches [25]. We propose that the main assumption of the WGCNA approach for clustering gene expression data can be used for HTG data. With that approach, we can detect highly interconnected SNP modules that may work cooperatively in a pathway, eliminating the multiple-testing problem of GWAS and giving new opportunities to analyse whole genome HTG data using a systems genetics approach. The main objective of this study was to develop a scale-free weighted genetic interaction network method using whole genome HTG data in order to detect biologically relevant genetic modules, pathways and potential genetic biomarkers for complex diseases and traits. We achieved this objective by modifying and extending the WGCNA method to suit genetic interactions/correlations derived from HTG data and by defining genome-wide module association tests (GMAT).

## Results

The WISH network method can be applied to HTG data using two different ways of detecting the interaction patterns between SNPs: 1) based on genomic correlations and 2) based on their epistatic interactions. The WISH network based on genomic correlation was applied directly to real data. The WISH network method based on epistatic interactions was tested first on simulated data and afterwards applied to real data. All results are presented here.

### Application of the WISH network method based on genomic correlations

**Figure 1:**
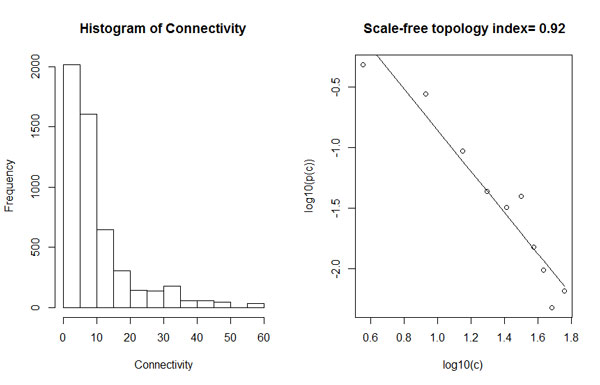
**Connectivity distributions to show scale-free topology**. The frequency distribution of the connectivity (left) shows a large number of low connected SNPs and a small number of highly connected SNPs. The log-log plot shows an R^2 ^(the scale-free topology index) of 0.92, which means the network is following the scale-free topology criterion.

**Figure 2:**
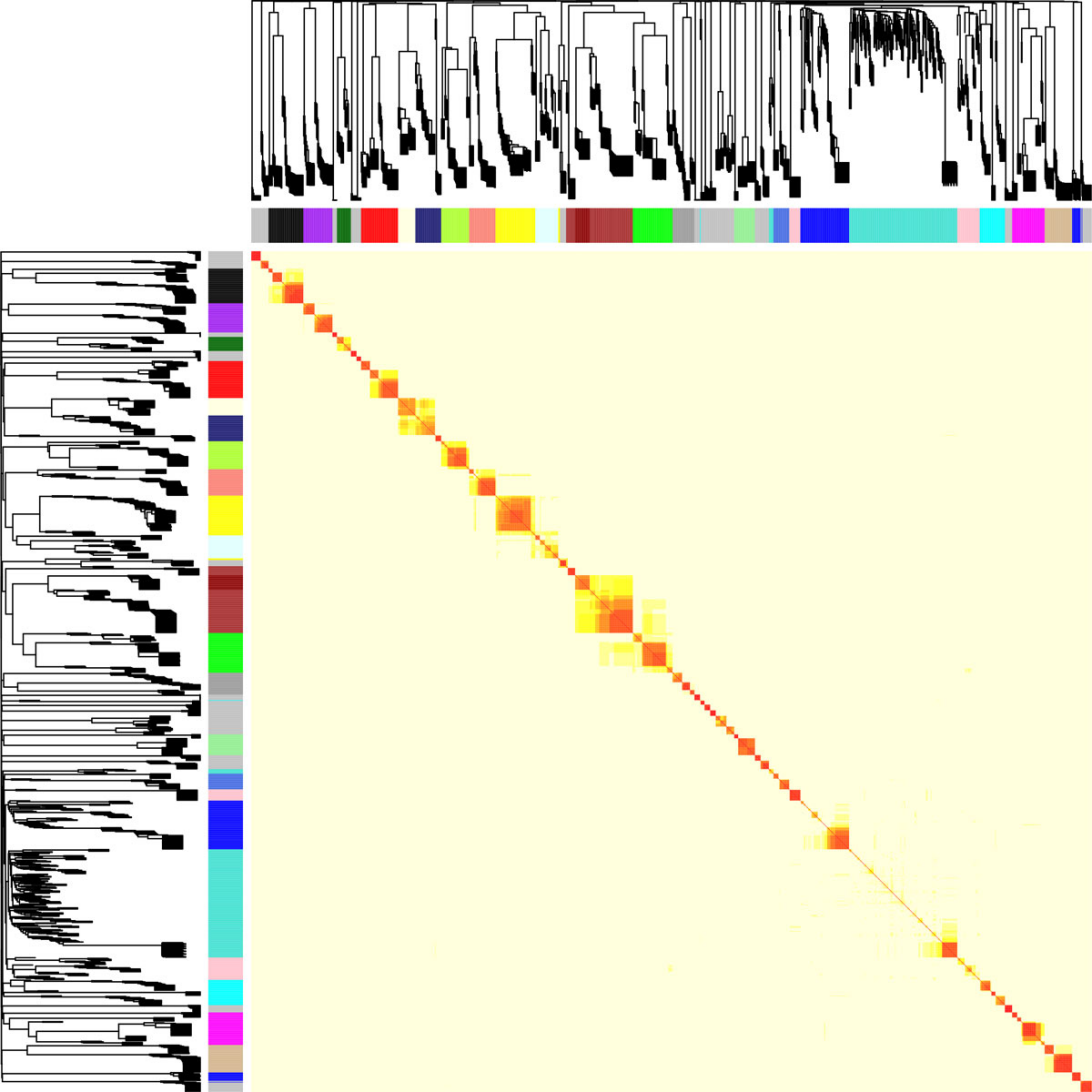
**Visualization of the network connections using a TOM plot**. Heat map of the Topological Overlap Measure (TOM) matrix. Genes are sorted by the SNP dendrogram (rows). Light yellow colour represents a low TOM, and a more red colour represents higher TOM. Clusters correspond to squares along the diagonal. The SNP dendrogram and module assignment by the Dynamic Tree Cut algorithm are shown along the left side and at the top.

The WISH method based on genomic correlations was tested using HTG data of an F2 pig resource population [26], with carcass weight as the trait of interest. The adjacency matrix (**A**) was created by calculating the correlations between the SNP genotypes and based on scale-free topology. The **A **matrix was raised to the power 5. Based on the connectivity of individual SNP with all the other SNPs in the network (Figure 1), the top 1500 highest connected SNPs were selected for the network construction. After SNP dendrogram was created, we detected 23 SNP modules each containing at least 30 SNPs based on Topological Overlap Measure (TOM) and the Dynamic Tree Cut method for dendrograms [27] (Figure 2).

**Figure 3:**
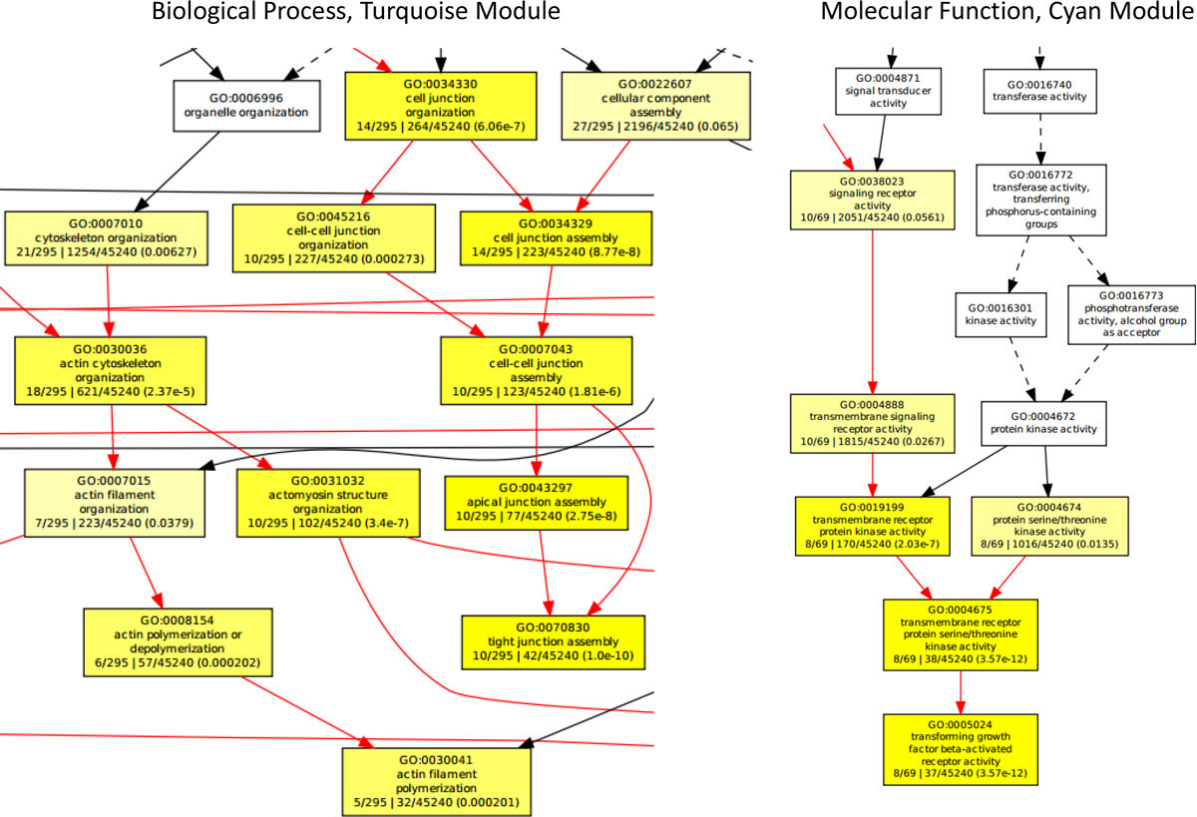
**Hierarchical tree graphs of highly enriched GO-terms**. Hierarchical tree graph of highly enriched GO-terms were constructed using GOEAST. The left graph shows the Biological Process in the Turquoise module, representing terms associated with muscling. The right graph illustrates the Molecular Function in the Cyan module, representing terms associated with cell growth.

For selection of biologically relevant modules, the Genome-wide Module Association Test (GMAT) was performed to detect potential biologically relevant modules, relating the module eigengenes to the complex trait observations on individual pigs (the genetic potential or EBVs for carcass weight). In total, three modules were selected for functional enrichment using the Gene Ontology Enrichment Analysis Software Toolkit (GOEAST) [28]: the Blue module (GMAT = 0.62, 62 SNPs), the Cyan module (GMAT = -0.62, 35 SNPs) and the Turquoise module (GMAT = 0.42, 171 SNPs). Genes present in the modules and corresponding pathways were determined and annotated using the NCBI2R R-package. In total, 54 SNPs (87%) of the Blue module, 32 SNPs (91%) of the Cyan module and 124 SNPs (73%) of the Turquoise module were present in or near one or several genes. Functional enrichment of the genes in the selected modules resulted in the identification of various pathways, which could be related to carcass weight. The majority of the detected pathways tended to be related to carcass weight. We also found highly enriched Gene Ontology (GO) terms associated with muscling, e.g., *actin filament processes *(Biological Process, Turquoise module), (cell) growth, e.g. *transforming growth factor (TGF) beta-activated receptor activity *(Molecular Function, Cyan module) and with fatness, e.g., *glucose catabolic process *(Biological Process, Blue module) (Figure 3). Identified significant GO terms using GOEAST are presented in Additional File 1. In conclusion, results of the WISH network analysis showed that the biologically relevant SNP modules and pathways associated with carcass weight could be detected.

### The WISH network method based on simulated epistatic interactions data

**Figure 4:**
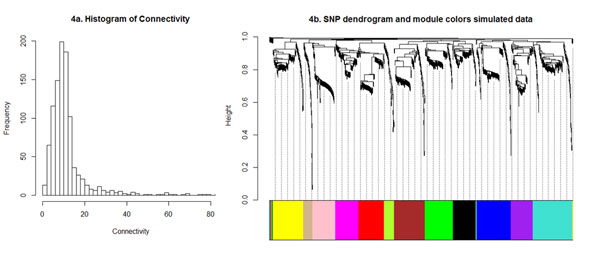
**Visualization of the simulation data**. The frequency distribution of the connectivity (Figure 4a) shows a large number of low connected SNPs and a small number of highly connected SNPs, which means the network is following the scale-free topology criterion. The SNP dendrogram (Figure 4b) shows clear clustering of the simulated SNPs and the colours represent the detection of modules (highly interconnected SNPs).

The WISH method could be improved by replacing the correlations between SNP genotypes with the actual epistatic interaction effects between SNP pairs. To test this assumption, we simulated pairwise epistatic interaction effects between 1000 SNPs based on distributional assumptions for epistatic interactions described in Cheverud et al. [29] which follows a normal distribution with values between -1 and 1. This is similar to estimated regression coefficients from a linear regression model, fitting a pairwise SNP_i _*x *SNP_j _interaction terms to phenotypes that are centered to have N (0,1) distribution. After raising the adjacency matrix to the power 4 (γ), the connectivity of the simulated data show that the network is scale-free (R^2 ^= 0.95) (Figure 4a). Using clustering of interconnected SNPs based on the dissimilarity **TOM**, ten modules were detected (Figure 4b). These results show that the WISH network method based on inputs from pairwise SNP *x *SNP epistatic interactions results in identification of "highly interactive" SNP modules underlying complex traits.

### The WISH network based on real epistatic interactions data

**Figure 5:**
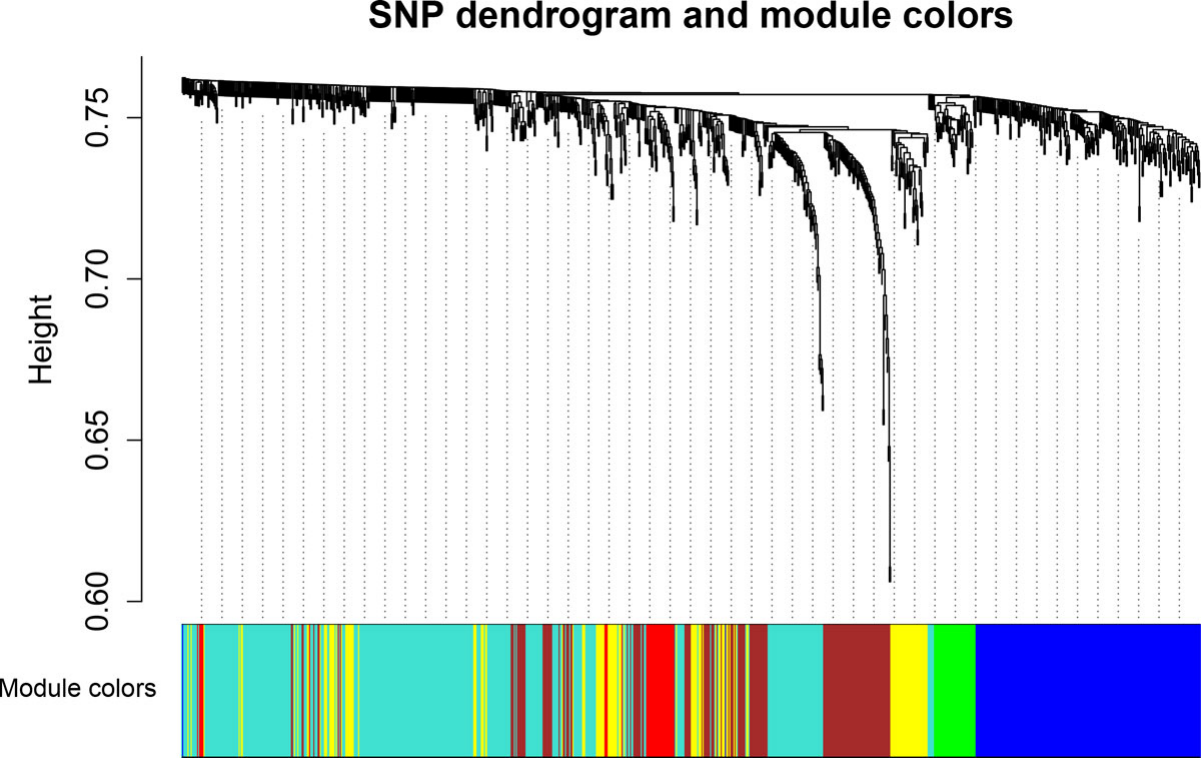
**Visualization of the WISH network based on epistatic interactions - real data**. The SNP dendrogram shows clustering of the SNPs (assigned to colours), using the genotype data from the F2 pig resource population with carcass weight as trait of interest.

The same F2 pig resource population and carcass weight data used in the WISH based on genomic correlations were also used to perform the WISH network method based on epistatic interactions. In total, 995 SNPs (top SNPs in the GWA study) were tested in an epistatic model and the regression coefficients of the SNP_i _*x *SNP_j _component were used as input for the WISH network. To test if the network is scale-free, the connectivity was calculated and the power γ of 4 was applied, resulting in the highest scale-free topology index: R^2 ^= 0.69. In total, five modules were created with at least 25 SNPs in each module (Figure 5). Out of five modules, one was appropriate for further downstream analysis based on their GMAT with the EBVs for carcass weight: the Red Module (GMAT = -0.41, 24 SNPs). Significant pathways in this module were detected using the NCBI2R R-package. Two pathways were significantly present in the Red Module: the *PI3K-Akt signalling pathway *(P_adj _= 2.43e-12) and the *synaptic vesicle cycle *(P_adj _= 1.40e-2) which have been associated with cell growth and insulin resistance.

## Discussion

Since 2008, several microarray gene co-expression studies have successfully used the WGCNA method to detect biologically relevant pathways [21,22,30,31]. We have presented an extension of the WGCNA method from using transcriptomic datasets to HTG datasets by developing the WISH network method. The WISH network method makes it possible to create a genetic interaction network and identify biologically relevant pathways using HTG data. WISH network method starts by reducing HTG data using the genome-wide significance and the variance of the SNPs in a certain population, for computational as well as biological reasons. Computationally, network construction is difficult, if we want to use all genome-wide markers on a SNP Chip (at present it could be up to a million in agricultural species and several millions in humans); therefore, a data reduction method is necessary. The number of SNPs used for network construction determines the size of the network and consequently the number of modules detected and the number of SNPs per module. Biologically, only those SNPs with acceptable genome-wide statistical significance would make any sense for further biological interpretation. Furthermore, SNPs with limited amount of genetic variation in the population were deselected, as they will make limited sense using a network approach. In rare cases, two SNPs which are individually not genome-wide significant may show a significant interaction effect, but they may be excluded from the analysis based on the significance threshold. This is a drawback of SNP selection because of computational limitations. However, we have minimised this problem by selecting all "marginally significant" SNPs whose significance levels were way below the genome-wide significance levels; hence we believe this is a valid selection criteria. Potentially, individuals can be selected for data reduction and to simultaneously increase the power of module detection. For instance, comparing two extreme phenotype groups will result in more extreme differences in interaction effects. We also constructed WISH network based on the interactions between SNP-pairs, estimated using real data in pigs as well as the simulated datasets. This showed the possibility to detect modules, clusters of highly interacting SNPs that are involved in creating complex trait variation. The modules detected by the WISH method were further analysed using functional enrichment analysis which led to the detection of biologically relevant pathways and potential genetic markers for carcass weight. Both methods, WISH based on genomic correlations and WISH based on epistatic interactions, result in the construction of a scale-free network. A large difference found between the two methods, was the number of identified modules (23 versus 5). We believe this is mainly due to the limited number of SNPs in the epistatic network to estimate the interaction effect, which limits the information of the clustering of SNPs. Both resulted in the identification of SNP *x *SNP interactions between and within chromosomes, and moreover, complement each other by finding different modules of biologically relevant modules.

An assumption made in gene co-expression studies is that similarly expressed genes over different treatment groups are functioning together in pathways. This assumption gives the possibility to reduce data, define disease subclasses and eventually identify pathways [32]. So far, possibilities for clustering using HTG data based on SNP *x *SNP interactions are not at all available, but the proposed WISH network method provides those opportunities. The critical assumption in WISH is that highly interconnected (interacting) SNPs are expected to be involved in similar genetic and molecular mechanisms affecting that phenotype. This is similar to the assumption made about gene expression values in WGCNA. Furthermore, fitting genome-wide genetic (SNP) interactions in standard GWA study models is difficult because of exponential increase in sample size requirement, statistical inference and computational issues [9]. Hence, our WISH network method could be seen as a post-GWA study method to unravel genome-wide genetic interactions and explain more underlying genetic variation. Previously, the Association Weight Matrix (AWM) [16] has also been proposed as a post-GWA study method. The AWM uses the allelic substitution effects per gene (or SNP) per trait. By calculating correlation between allelic effects of SNPs between two traits, AWM re-generates polygenetic correlations between traits and by clustering allelic effects of all SNPs across phenotypes, AWM creates pleiotropic genetic network. WISH network aims and methods are different to AWM. First, WISH is not dependent on the estimation of the allelic substitution effects from GWA study and second WISH is intended to build genetic networks within one complex trait but not across multiple traits. The WISH network method only uses the genotype data, or the epistatic interaction data on one phenotype, to create an interaction network, in contrast to the AWM which uses the GWA study results on several traits to detect the network of SNPs that are pleiotropic (act across multiple traits). The AWM method is not weighted, nor does it follow scale-free topology. This means that WISH network construction and module detection is done without using prior knowledge. Hence, the WISH network method is hypothesis-free and will provide the opportunity for new insights in complex diseases and traits. At a later stage, the network can be related to the phenotype(s) to detect potential biologically relevant modules. To reveal the biological relevance of detected modules, analysis is largely dependent on existing functional annotation, which could be a limiting factor. On the other hand, the identification of pathways could also help us further in the functional annotation of SNPs and genes.

The WISH network method detects clusters of highly interacting SNPs. Previous studies have indicated that the structure of haplotypes, clusters of loci on the chromosome that are transmitted together, can increase the power of GWA studies. However, haplotypes only take the "correlations" between loci into account (e.g. r^2^) which are physically attached to each other. However, previous research has shown that linkage disequilibrium (LD) can also be measured across different chromosomes, and may explain more genetic variation in complex, polygenic phenotypes [33,34]. The WISH method detected interactions of SNPs across different chromosomes (inter-chromosomal LD) and was not limited by the physical location of the SNPs. The involvement of SNPs from various chromosomes was seen in all detected modules and was very clear in the Turquoise Module (WISH based on genomic interactions), which consisted of 124 SNPs spread over 17 chromosomes. This distant physical locations of SNPs in SNP modules created by the WISH method shows that it detects interactions along the whole genome.

We tested the WISH network method on an F2 pig resource population with carcass weight as the trait of interest. For this trait, we also performed a GWA study, using the R-package GenABEL. This resulted in the discovery of eight genome-wide significant SNPs, three of which were located on chromosome 2 (two physically close to each other), three on chromosome 7 (all physically close to each other), one on chromosome 1 and one on chromosome 4. Using NCBI2R, three genes were detected: KIAA0247 (a protein coding gene with unknown function), KLC2 (Kinesin Light Chain 2, a protein coding gene) and LOC100518234 (Breast cancer metastasis-suppressor 1 homolog). As a result of functional annotation, only the KLC2 gene can be related to biological function in relation to carcass weight, as it is known that kinesin acts as a molecular motor generating ATP-dependent movement of vesicles and organelles along the microtubules. In total the WISH network method detected three SNP modules that were significantly associated with carcass weight. After functional annotation, we found some pathways which could be biologically related to carcass weight, e.g., *actin filament processes *and *TGF beta-activated receptor activity*. Nevertheless, the KLC2 gene was not found in one of these modules, neither in the WISH based on genomic correlations nor in the WISH based on epistatic interaction. This implies that the WISH network method can be used as a supplementary analysis, detecting different SNPs for the trait of interest. Whereas a GWA study detects single SNPs, the WISH method detects groups of highly interconnected SNPs which are often present in the same pathway. Besides carcass weight, we tested the WISH network method on several other traits present in the F2 pig resource population [see trait information in Kogelman et al. [35]], and they all revealed biologically relevant results (results not shown).

Although we have shown the creation of one network using one single phenotype, several other ways of analysing phenotypes are possible using the WISH method. As proposed by the founders of WGCNA, a differential network can be created by comparing, for example, case and controls, using the same input data (same SNP data matrix), but creating two networks using two groups of individuals (e.g. case-control, different disease states). Several measurements, such as differential connectivity, can be used to identify modules with a different SNP effect profile between the two groups. The WISH network method can also be used as a multi-trait network approach. After network creation and module detection, the GMAT of detected modules with other (correlated) phenotypes can be calculated. By comparing the relatedness of modules with different phenotypes, the WISH network method could possibly be used to identify endophenotypes and subsequently pathways of endophenotypes of complex diseases.

The WISH network method based on epistatic interactions is theoretically a promising approach to cluster SNPs based on their interconnectedness. It has been shown in the simulation data that the epistatic data follows scale-free topology and clear clusters of highly interconnected SNPs are detected. However, the use of real data did not represent a clear scale-free network and consequently less clear modules are detected with lower correlations with the trait of interest. This could be due to several reasons. First, the number of SNPs in the network limits the size of the network. In the WISH based on genomic correlations the connectivity is measured over > 5000 SNPs and the most highly connected SNPs are concordantly selected for network construction. Because of computational and time limitations, we used only the top 1000 SNPs for the WISH network based on epistatic interactions. Secondly, we used all individuals present in the dataset to estimate the epistatic interaction effect to increase the power. However, the WISH network based on genomic correlations is created based on extreme phenotypes (EBVs). This will result in more extreme interaction effects; however, due to a limited number of individuals in our dataset, this was not possible in this study.

The WISH network method was tested on real data, using an F2 pig resource population, and was able to detect biologically relevant pathways using HTG data. We propose the WISH network method as a new systems genetics tool to investigate HTG data in order to improve understanding of the biological and genetic background of complex diseases and traits.

## Conclusions

We report here the development and application of a new systems genetics tool - the WISH network method - for building networks using HTG data obtained from genetic study populations. The basic principle of WISH network method is based on the existing WGCNA method for building co-expression networks using transcriptomic data. This study addresses the long gap or a need in studying genome-wide genetic interactions in the context of genetic (SNP) networks underlying complex trait variation. The proposed WISH network method has been tested using simulated datasets as well as applied to real experimental data obtained from a pig resource population, genotyped using 60K Porcine SNP Chip and extensively phenotyped for obesity and obesity-related traits. Results have shown that WISH detects biologically relevant modules for carcass weight (results shown) and other obesity-related traits (results not shown). The WISH network method can be used in human genetics to detect pathways of complex diseases as well as in animal and plant genetics to unravel the genetic background and detect genetic biomarkers of economically important production traits. By analysing various traits within the same network, the WISH network method is able to detect endo-phenotypes which could be valuable for treatment of individuals with complex diseases. Moreover, a differential WISH network method can be used in a case-control design to identify conserved pathways in the different groups or to identify pathways that behave differentially between the cases and controls.

## Materials and methods

**Figure 6:**
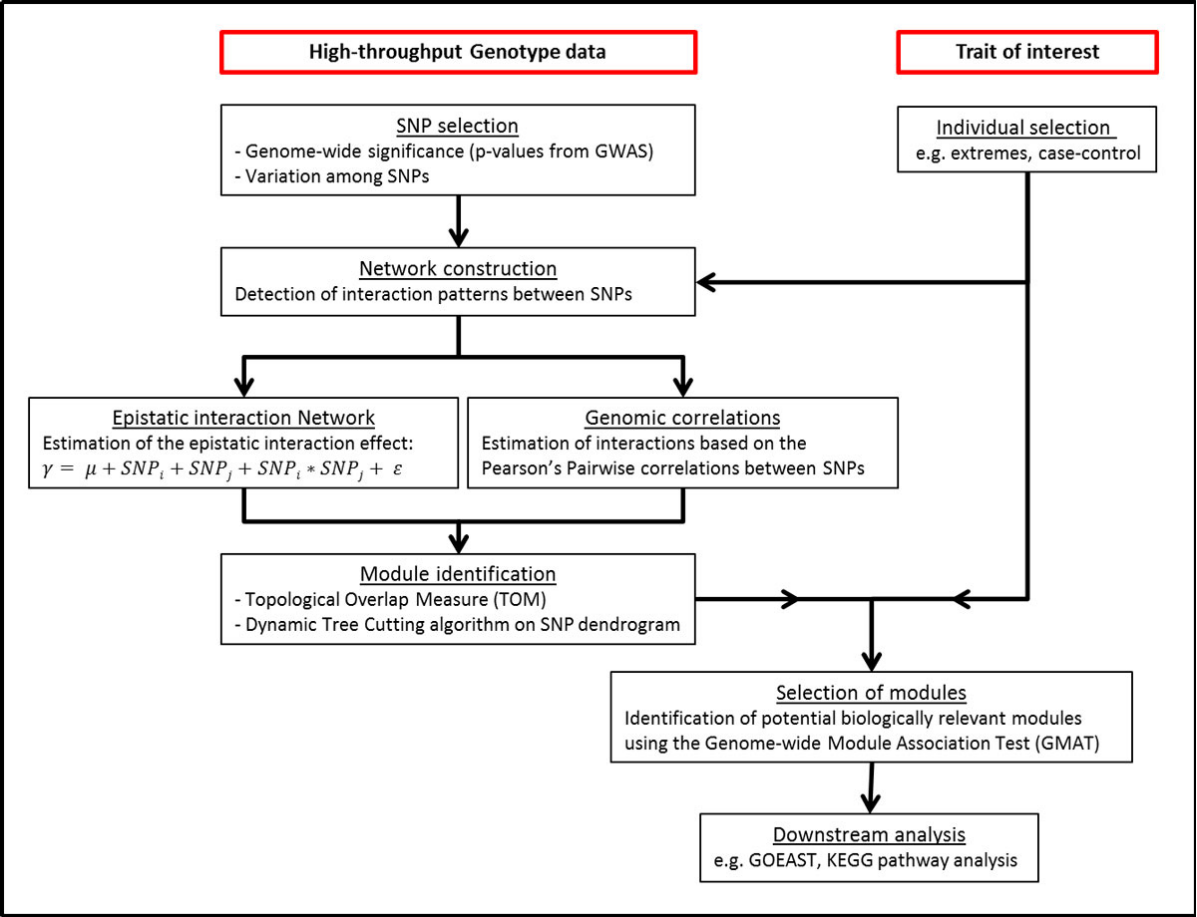
**WISH Workflow**. A workflow representing the steps involved in the WISH network construction.

The WISH network method is an extension of the R-package WGCNA [36] using HTG data from SNP genotyping arrays instead of gene expression data from microarray studies (Figure 6). The WGCNA method relies on the assumption that genes with correlated expression levels in given treatment or biological state in a group of individuals or replicates are functioning cooperatively in pathways, contributing to the condition or trait of interest. We extended this assumption to SNPs and assume that SNPs which are highly correlated (or interacting) among each other are functioning cooperatively and similarly in pathways.

### Data reduction

For computational reasons not all genome-wide markers on a SNP Chip can be used and therefore a data reduction step is necessary. Also, only those SNPs that have an acceptable (but not very stringent) genome-wide statistical significance would make any sense for further biological interpretation, and SNPs without a reasonable amount of genetic variation provide little information in a network setting. Hence, we select a number of top SNPs fulfilling both criteria for WISH network construction; 1) reaching a genome-wide significant p-value and 2) possessing sufficient amount of variation among the individuals in the population, based on their genotype. Depending on the size of the dataset, individuals can be optionally selected based on extreme phenotypic values.

### WISH network method based on genomic correlations

The WISH network describes the relationships between SNPs by specifying an *n *x *n *dimensional adjacency matrix **A **= A_ij_, where A_ij _states the connection strength between SNP_i _and SNP_j_. The connection strength between the SNPs is defined by the absolute Pearson's correlation between the number of allele copies of pairs of SNPs for all SNP pairs in the data, creating a weighted network with values for A_ij _between 0 and 1. This adjacency matrix is raised to the power *γ *(soft thresholding). The connectivity of a SNP (*c*) can concordantly be calculated by taking the sum of connection strengths between a SNP and all other SNPs. SNPs with a high *c *can be called hub SNPs, which may be of biological importance to the trait of interest [22,37]. A WISH network follows scale-free topology, meaning that the network will consist of a few highly connected SNPs (hub SNPs) and many low connected SNPs. In the case of a scale-free network, the frequency distribution of *c *(*p(c)*) follows a *power law*. The power *γ *should be chosen in such a way that R^2 ^(the scale-free topology index) approaches one, which means the network is approximately scale-free [38]. The assumption of scale-free topology can be tested by plotting *p(c) *and by plotting *log(p(c)) *versus *log(c)*. Optionally, data can be further reduced by using a threshold value for selection of SNPs based on *c*, to eliminate the SNPs with an extremely low *c*.

The adjacency matrix is used to detect the relatedness between pairs of SNPs based on their shared neighbours by calculating the Topological Overlap Measure (TOM) [39,40].

$${TOM_{i}}_{j}=\frac{{{\text{N}}_{i}}_{j}+{\text{A}}_{ij}}{\min\left\{c_{i},c_{j}\right\}+1-{{\text{A}}_{i}}_{j}}$$

where N_ij _is the number of SNPs to which both SNP_i _and SNP_j _are connected, A_ij _is the adjacency between SNP_i _and SNP_j_, and *c *is the connectivity. The TOM of two SNPs represents the number of shared neighbour-SNPs as a value between 0 and 1; here a TOM of 1 means that a pair of SNPs shares the same neighbour-SNPs and a TOM of 0 means that a pair of SNPs shares no neighbour SNPs at all. The TOM is resulting in a more robust network in the case of many zeroes among the elements in the adjacency matrix or when elements are prone to noise [41]. Using the dissimilarity **TOM **(1-**TOM**), a SNP dendrogram was created whereby branches of the dendrogram were cut off by the Dynamic Tree Cut algorithm [27] representing the SNP modules (clusters of highly interconnected SNPs). Each module was assigned to a colour. By plotting the **TOM **as a heat plot, modules can be visualized, and the right cut-off method can be chosen.

A module is defined as a cluster of highly interconnected SNPs, forming a sub-network which may correspond to a pathway or protein complex, resulting in substantial practical importance in biology. A second visualization to control the detection of modules by the chosen cut-off method is plotting the correlations between module eigenSNPs. The module eigenSNP is the first principal component of the module and can be seen as a representative effect of the module on the trait of interest. Optionally, modules with a strong correlation can be merged. Secondly, the module eigenSNP can be used to examine the relationship between the trait of interest and the module, resulting in a Genome-wide Module Association Test (GMAT) to identify which modules are relevant for downstream analyses.

After detection of the potentially interesting SNP modules via GMAT, genes in the SNP modules have to be detected. To detect the positions of the SNPs and the genes present in those regions, we used the R-package NCBI2R (available at http://cran.r-project.org/web/packages/NCBI2R/index.html) with default parameters which uses a list of SNPs as input and gives (if present) the genes and their annotation in the indicated region. The flanking distance could be changed depending on the species used in the study. In the next step, the genes in those SNP lists can be used for functional annotation analyses, for example using the Gene Ontology Enrichment Software Toolkit (GOEAST) with default parameters, which will reveal if identified SNP modules are biologically relevant to the trait of interest. GOEAST detects highly enriched GO-terms in the genes present in the SNP modules and defines highly enriched pathways, which can help to understand the results biologically. Enriched GO-terms with a p-value (estimated by a hypergeometric test) below 0.05 were assigned to be significant.

### WISH network method based on epistatic interactions

**Figure 7:**
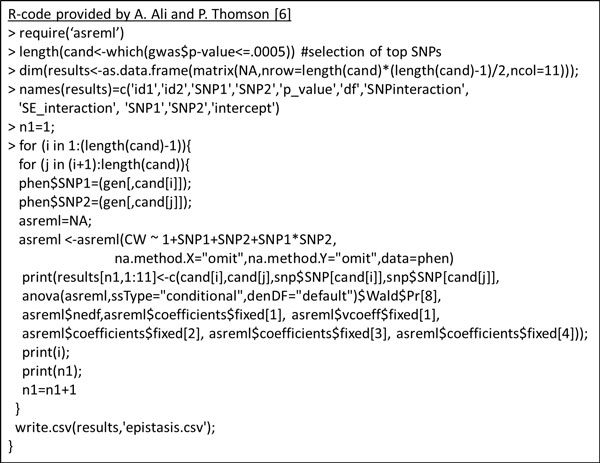
**R-codes for the WISH network based on epistatic interactions**. The epistatic interaction effect between SNP pairs can be calculated using these R-functions, provided by A. Ali and P. Thomson [6]

The second option for creating a network using HTG data is creating an adjacency matrix based on the epistatic interactions. We used a linear model to estimate the regression coefficient for the epistatic (SNP_i_*SNP_j_) interaction:

$$\gamma =\mu +\text{SN}{\text{P}}_{\text{i}}+\text{SN}{\text{P}}_{\text{j}}+\text{SN}{\text{P}}_{\text{i}}*\text{SN}{\text{P}}_{\text{j}}+\varepsilon$$

where *γ *is the trait of interest or a trait pre-adjusted for systematic environmental effects, *μ *is the intercept, SNP_i _and SNP_j _is the genotype coded as 1/1.5/2 (1 = 0 minor alleles copies, 1.5 = 1 minor allele copy, 2 = 2 minor allele copies), SNP_i_*SNP_j _is the interaction between the pair of SNPs as a covariate, and *ε *is the residual component. This analysis was performed using the R-package ASReml-R [26]. In total, four data frames were needed to run the epistatic model: 1) *snp*: SNP information (3 columns: SNPname, chromosome, position), 2) *gwas*: GWAS results, e.g. from GenAbel, 3) *phen*: data frame with one column for the individual ID, one column for the sex, and afterwards the columns for the phenotypes and/or fixed effects, and 4) *gen*: a genotype matrix of *m*-by-*n *with *m *the number of animals and *n *the number of SNPs. R-codes using those four data frames are provided in Figure 7.

The regression coefficient resulting from the SNP_i_*SNP_j _component is used as input for the adjacency matrix. The regression coefficients are normalized to create a data matrix with values between 0 and 1. From here on, the methods are comparable to the methods used in the WISH based on genomic correlations. First, we detected which power γ results in the highest scale-free topology index (R^2^) and we raised the adjacency matrix to this power. Secondly, the TOM was calculated and the dissimilarity-TOM was used to create a SNP dendrogram. Modules were again detected using the Dynamic Tree Cut algorithm. Thirdly, potential biologically relevant modules were detected by calculating the GMAT. For the calculation of the module eigengenes, the genomic matrix of *m*-by-*n *was used to calculate the module eigengene per module, per individual. Modules with a significant GMAT above a certain threshold were selected for downstream analysis. At last, genes and KEGG-pathways in the modules were detected using the R-package NCBI2R, where KEGG-pathways with a Bonferroni corrected p-value below 0.05 were assigned to be significant.

### Materials

Phenotype and genotype data (60K Porcine SNP Chip) of an F2 pig resource population were available to test and assess the WISH network on real data [35], and in this study we selected carcass weight at slaughter as phenotype of interest to test the method. Carcass weight had an estimated heritability of 0.54 (SE = 0.14), which indicates a high degree of genetic variation for this trait in the pig resource population. After adequate quality control measurements, the GWA study was performed using 39,704 SNPs using the R-package GenABEL[42]. Based on the GWA results, SNPs were selected on the basis of their genome-wide significance. One dataset was created with one genotype value per SNP, by calculating the average of the 2 alleles (1,2-coding). Secondly, in concordance with WGCNA, which selects genes based on their variance, the most varying SNPs in the population were selected due to computational limitations. In the WISH network based on genomic correlation, a total of 5219 SNPs and 75 animals from the Duroc*Göttingen Minipigs based on their estimated breeding values (EBVs) for carcass weight (25 high, 25 intermediate, and 25 low) were selected for network construction. In the WISH network based on epistatic interactions a stricter threshold had to be chosen for the selection of SNPs because of computational limitations, resulting in 995 SNPs. All animals from the Duroc*Göttingen Minipigs population were used for the estimation of epistatic interactions to increase the power.

## Authors' contributions

HNK conceived the original idea of WISH networks and GMAT. LJAK and HNK developed the WISH methodology and LJAK implemented the methodology on experimental data. LJAK wrote the first draft of the manuscript. Both authors wrote, read, and approved the final version of the manuscript.

## Competing interests

The authors declare that they have no competing interests.

## Supplementary Material

Additional file 1 GOEAST results on carcass weight (based on genomic correlations) Click here for file

## Declarations

The authors would like to thank Abdirahman A. Ali and Peter C. Thomson for their help in the R-codes for the epistasis association model to estimate interaction regression coefficients. We thank Merete Fredholm for testing our method on collected phenotype data on the pig population and the BioChild consortium http://www.biochild.ku.dk for the use of the high-throughput genotype data on those animals. We thank Sameer D. Pant for useful discussions. Lisette J. A. Kogelman was financially supported by a PhD stipend awarded to her by the University of Copenhagen. Haja N. Kadarmideen is a recipient of the EU-FP7 Marie Curie Actions - Career Integration Grant (CIG-293511) which partially funded this work. The publication cost of this article was partially supported by COST-BMBS, Action BM1006 "Next Generation Sequencing Data Analysis Network", SeqAhead.

This article has been published as part of *BMC Systems Biology *Volume 8 Supplement 2, 2014: Selected articles from the High-Throughput Omics and Data Integration Workshop. The full contents of the supplement are available online at http://www.biomedcentral.com/bmcsystbiol/supplements/8/S2.
